# Human Intestinal Enteroids to Evaluate Human Norovirus GII.4 Inactivation by Aged-Green Tea

**DOI:** 10.3389/fmicb.2020.01917

**Published:** 2020-08-18

**Authors:** Walter Randazzo, Veronica Costantini, Esther K. Morantz, Jan Vinjé

**Affiliations:** ^1^Division of Viral Diseases, National Calicivirus Laboratory, Centers for Disease Control and Prevention, Atlanta, GA, United States; ^2^Department of Microbiology and Ecology, University of Valencia, Valencia, Spain; ^3^Cherokee Nation Assurance, Arlington, VA, United States

**Keywords:** aged-green tea, human norovirus, human intestinal enteroids, Tulane virus, natural compound

## Abstract

Human noroviruses are the leading cause of epidemic and sporadic acute gastroenteritis worldwide and the most common cause of foodborne illness in the United States. Several natural compounds, such as aged-green tea extract (aged-GTE), have been suggested as ingestible antiviral agents against human norovirus based on data using murine norovirus and feline calicivirus as surrogates. However, *in vitro* data showing their effectiveness against infectious human norovirus are lacking. We tested the activity of aged-GTE to inhibit human norovirus in a human intestinal enteroids (HIEs) model and Tulane virus in LLC-monkey kidney (LLC-MK2) cell culture. HIE monolayers pretreated with aged-GTE at different temperatures showed complete inhibition of human norovirus GII.4 replication at concentrations as low as 1.0 mg/ml for 37°C, 1.75 mg/ml for 21°C, and 2.5 mg/ml for 7°C. In contrast, a moderate decrease in Tulane virus infectivity of 0.85, 0.75, and 0.65 log TCID_50_/ml was observed for 2.5 mg/ml aged-GTE at 37, 21, and 7°C, respectively. Our findings demonstrate that GTE could be an effective natural compound against human norovirus GII.4, while only minimally effective against Tulane virus.

## Introduction

Human noroviruses are the leading cause of epidemic and sporadic acute gastroenteritis worldwide and the most common cause of foodborne illness in the United States and Europe, with a societal cost of $60 billion dollars (Ahmed et al., 2014; Havelaar et al., 2015; Bartsch et al., 2016). The global incidence of human noroviruses transmitted through food has been estimated as 120 million cases and 35,000 deaths (World Health Organization, 2018). In spite of underreporting, the last available official report from 2017 lists human norovirus among the most reported causative agent in foodborne outbreaks, accounting for 140 outbreaks (35% of all outbreaks) in the United States and 211 outbreaks (7.8%) in Europe (Pires et al., 2015; Centers for Disease Control and Prevention, 2017; EFSA, 2018).

Norovirus infections are difficult to prevent due to their low infectious dose, high shedding titer, and environmental stability. The virus can spread through multiple transmission routes, even though the most common are the person-to-person and the foodborne transmissions. Most norovirus outbreaks are reported in long-term care facilities, schools, hospitals, restaurants, and cruise ships.

Currently there are no licensed norovirus vaccines available (Mattison et al., 2018) and control efforts rely on promoting and adhering to hand hygiene practices, enhanced environmental cleaning and disinfection, exclusion of sick staff from work, ward closures, and cohorting sick patients (Barclay et al., 2014).

Several antiviral molecules have shown to inhibit norovirus replication by preventing either the viral binding to cell receptor (i.e., dimethyl cyclopenta-a phenanthren analogues), the maturation of viral proteins (i.e., aldehyde inhibitors and Michael-acceptor polypeptide inhibitors), or the replication of virus genome (i.e., ribavirin, favipiravir, and suramin; Rocha-Pereira et al., 2014).

Among natural extracts, green tea extract (GTE), a derivative of the cultivated evergreen tea plant (*Camellia sinensis* L.), is rich in polyphenols and has demonstrated antimicrobial activity against a wide range of pathogens such as Zika virus, dengue virus, and foodborne pathogens (Perumalla and Hettiarachchy, 2011; Steinmann et al., 2013). GTE has been shown to inhibit enteric virus replication, including hepatitis A virus (HAV), murine norovirus (MNV), and feline calicivirus (FCV; Seo et al., 2016; Randazzo et al., 2017; Falcó et al., 2019). Interestingly, controlled storage conditions (24 h at 25°C) significantly increased GTE antiviral activity, likely due to the increasing accumulation of catechin derivatives (Falcó et al., 2018).

In the absence of a robust *in vitro* cell culture system for human noroviruses (Moore et al., 2015; Manuel et al., 2018), antimicrobial activity of GTE against these viruses has been inferred by quantitative reverse transcription polymerase chain reactions (RT-qPCRs) and capsid binding assays as indirect approximations (Falcó et al., 2019). Only very recently, a novel three-dimensional (3D) cell culture technique based on non-transformed stem cell-derived human intestinal enteroids (HIEs), which recapitulate the complexity and cell diversity of the gastrointestinal tract has been reported (Ettayebi et al., 2016; Costantini et al., 2018; Chan et al., 2019).

Tulane virus has been used as a cultivable surrogate virus for human norovirus to assess the efficacy of disinfection products and methods. Compared to other surrogate viruses such as FCV and MNV, Tulane virus is one of the most promising to use experimentally, as this virus is an enteric virus and displays diverse histo-blood group antigen (HBGA) binding patterns similar to human noroviruses (Cromeans et al., 2014; Farkas, 2015; Polo et al., 2018). In this study, we evaluated the antiviral activity of GTE against GII.4 norovirus in HIEs and Tulane virus in LLC-monkey kidney (LLC-MK2) cells.

## Materials and Methods

### Green Tea Extract and Viruses Used in the Study

GTE (Naturex SA, France) was dissolved in PBS (pH 7.2) to obtain a concentration of 10.00 mg/ml. The GTE solution was aged for 24 h at room temperature as reported previously (Falcó et al., 2017, 2018; Randazzo et al., 2017). Fecal filtrates from GII.4 Sydney[P16] and GII.4 Sydney[P4 New Orleans] human norovirus-positive fecal samples were prepared by passing clarified stool extracts through four serial filters (5, 1, 0.45, and 0.22 μm) as described previously (Costantini et al., 2018) and stored at −70°C until the time of testing. Tulane virus was grown in LLC-MK2 cell line (ATCC CCL-7) cultured in Opti-MEM (Gibco Life Technologies, Grand Island, NY) supplemented with 2% FBS and 1% antibiotic-antimycotic cocktail (STR/Pen; Life Technologies Corporation, Carlsbad, CA), as described previously (Cromeans et al., 2014; Esseili et al., 2018).

### Maintenance and Differentiation of Human Intestinal Enteroids

Three-dimensional HIEs were derived from human jejunal biopsies (J3 cell line) and maintained as described previously (Costantini et al., 2018). Briefly, HIE cultures were grown as undifferentiated 3D cultures in the presence of IntestiCult™ Organoid Growth Medium (Human; STEMCELL™ Technologies; referred to as CMGF− medium) supplemented with 10 μmol/l Y-27632. Undifferentiated monolayers were produced by plating single cell suspensions obtained from 7 days old highly dense 3D cultures. After 24 h at 37°C and 5% CO_2_, differentiation of the HIE was initiated by replacing IntestiCult™ Organoid Growth Medium (Human; STEMCELL™ Technologies) with differentiation medium.

### Cytotoxicity of Aged-GTE on HIE and LLC-MK2 Cells

To assess potential cytotoxicity of aged-GTE on HIE and LLC-MK2 cells, cells were incubated with different concentrations of aged-GTE (up to 2.5 mg/ml) stained with trypan blue and visually inspected for cytotoxicity by using a Zeiss Axiovert 200 microscope. The proportion of live cells was determined using a Countess™ II cell counter (Thermo-Fisher). Moreover, the viral replication capability of HIEs was assessed by infecting monolayers pretreated with aged-GTE with human norovirus and measuring virus replication by RT-qPCR.

### Antiviral Treatment and HIE Infection

Aged-GTE was diluted in CMGF− medium to 0.10, 0.40, 0.80, 1.00, 1.75, 2.50, and 5.00 mg/ml. Clarified human norovirus-positive stool suspensions (GII.4 Sydney[P16]: 4.62 ± 0.2 log_10_ gc/100 μl and GII.4 Sydney[P4 New Orleans]: 4.16 ± 0.0 log_10_ gc/100 μl) were mixed 1:1 v/v with aged-GTE, incubated 1 h at 37, 21, and 7°C and neutralized by mixing with an equal volume of CMGF−. CMGF− medium alone, virus and CMGF, and virus without aged-GTE were included as negative controls. To determine viral infectivity, two sets of 96-well plates with 100% confluent 4–6 day-old-differentiated HIE monolayers were inoculated in triplicate with 100 μl of aged-GTE-treated human norovirus samples and incubated at 37°C for 1 h. After the inoculum was removed, monolayers were washed twice with CMGF− medium and 100 μl of differentiation medium containing 500 μM GCDCA and 50 μmol/l ceramide was added to each well. For each set of infections, one 96-well plate was immediately frozen at −70°C (1 hpi) and the second plate was incubated at 37°C and 5% CO_2_ for 24 h and then frozen (24 hpi).

### Extraction, Detection, and Quantification of Human Norovirus

Viral RNA was extracted from HIE monolayers at 1 and 24 hpi using the KingFisher instrument and MagMAX-96 Viral RNA Isolation Kit (Ambion, Austin, TX, USA) according to the manufacturer’s instructions. Norovirus RNA was detected by GI/GII TaqMan RT-qPCR (Cannon et al., 2017). A 10-fold serial dilution of a quantified 3 kb GII.4 norovirus RNA transcript was included in each experiment to quantify the RNA into genome equivalents.

### Infectivity, Extraction, and Detection of Tulane Virus

Tulane virus (4.02 ± 0.2 log_10_ TCID_50_/ml or 4.76 ± 0.02 genomic copies/ml) was exposed to aged-GTE at final concentrations ranging from 0.05 to 2.5 mg/ml for 1 h at 37, 21, and 7°C and neutralized by mixing with an equal volume of Opti-MEM. An untreated sample (media only) was included as negative control. Then, 10-fold serial dilutions of Tulane virus were prepared in Opti-MEM and inoculated on eight wells with 100% confluent LLC-MK2 monolayers in 96 well plates and 20 μl of aged-GTE-treated Tulane virus per well. After incubation for 1 h at 37°C, 180 μl Opti-MEM plus 2% FBS was added. After 3–5 days, cells showing cytopathic effect (CPE) were enumerated and the Tulane virus titer was calculated as log_10_ (*N*_x_/*N*_0_), where *N*_0_ is the infectious virus titer for untreated samples and *N*_x_ is the infectious virus titer for aged-GTE-treated samples. To determine effect of aged-GTE on the viral genome, Tulane virus RNA was extracted as described above and detected by RT-qPCR (Chhabra et al., 2017). Genome copies were calculated by using a standard curve of 10-fold serial dilutions of a purified 4 kb PCR product amplified from the 3'-end of the virus genome.

### Statistical Analysis

All data were compiled from three independent experiments with three technical replicates for each variable. Data are presented as mean ± SD. Significant differences in mean infectivity were determined by using one-way ANOVA followed by Dunnett’s multiple comparisons test. Differences in means were considered significant when the *p* was <0.05. The nonlinear regression (curve fit) function from GraphPad Prism version 7 (GraphPad Software, USA) was used to obtain EC_50_ values.

## Results

### Cytotoxicity of Aged-GTE

**Figure 1:**
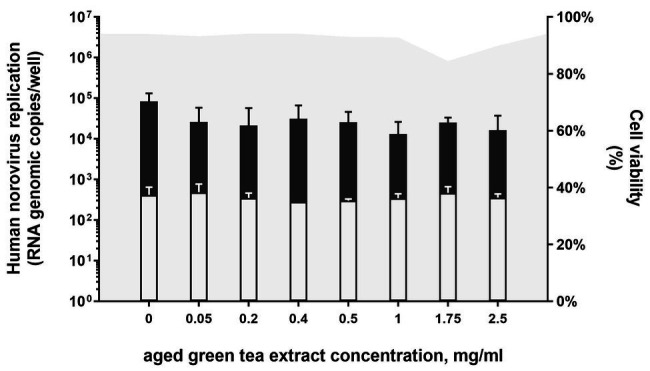
Human norovirus replication on HIEs pretreated with aged-green tea extract (GTE; bars) and related cell viability (gray area). HIEs were treated with aged-GTE at concentrations up to 2.5 mg/ml for 1 h at 37°C. Aged-GTE pretreated HIEs were counted by an automated cell counter to check for viability and reported as percentage of viable cells. Aged-GTE-pretreated HIEs were infected with human norovirus suspensions (GII.4 Sydney[P16] and GII.4 Sydney[P4 New Orleans]) and results are reported as log increase in human norovirus RNA per well at 24 hpi (black bars) compared with 1 hpi (white bars). Data represent means and error bars indicate SDs of three experiments with three wells for each treatment and time point. Dotted lines represent quantitative reverse transcription polymerase chain reaction (RT-qPCR) limit of detection.

First, microscopical visual inspection of HIEs monolayers exposed to GTE for 1 h at 37°C in 5% CO_2_ showed no cytotoxic effect. Similarly, no differences on cell viability were detected for samples treated with aged-GTE at concentrations up to 1 mg/ml (mean cell viability 94%), while slight decreases were observed at 1.75 and 2.5 mg/ml (Figure 1). Human norovirus replication on HIEs pretreated with 0.05, 0.2, 0.4, 0.5, 1.0, 1.75, and 2.5 mg/ml aged-GTE showed no statistical differences on viral titers at 24-h post-infection (hpi; *p* = 0.266; Figure 1).

Similarly, LLC-MK2 cells used to test Tulane virus showed no cytotoxic effect for aged-GTE at testing concentrations as resulted from visual inspection (data not shown).

### Aged-GTE Inhibits Viral Replication

The human norovirus initial concentrations and the control titers (untreated sample, 0 mg/ml aged-GTE) at 24 hpi were consistent across all experiments, allowing the comparison among GTE treatments and exposure temperatures.

**Figure 2:**
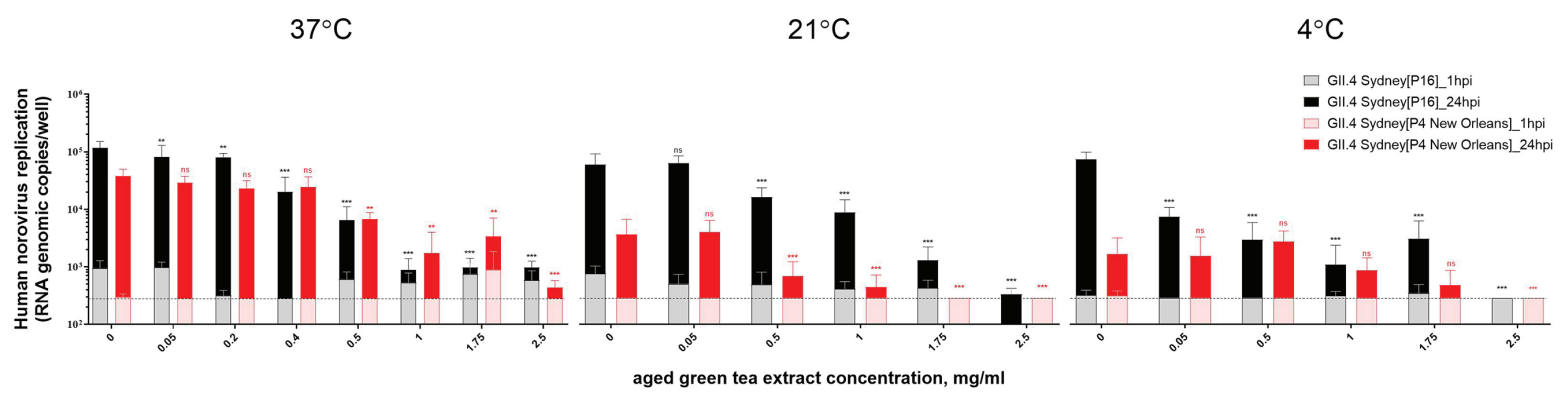
Inactivation of human norovirus with aged-green tea extract (GTE) in suspension. Human norovirus suspensions (GII.4 Sydney[P16] or GII.4 Sydney[P4 New Orleans]) were treated with aged-GTE at concentrations up to 2.5 mg/ml for 1 h at 37, 21, and 7°C. The mean of each data point is shown and error bars indicate SDs of three experiments with three wells for each treatment and time point. Dotted lines represent RT-qPCR limit of detection. For each sample, we performed one-way analysis of variance followed by Dunnett’s multiple comparisons test. *p* is compared with the nontreated sample: ^**^p < 0.01; ^***^p < 0.001; ns, not significant. hpi, hours postinfection.

**Figure 3:**
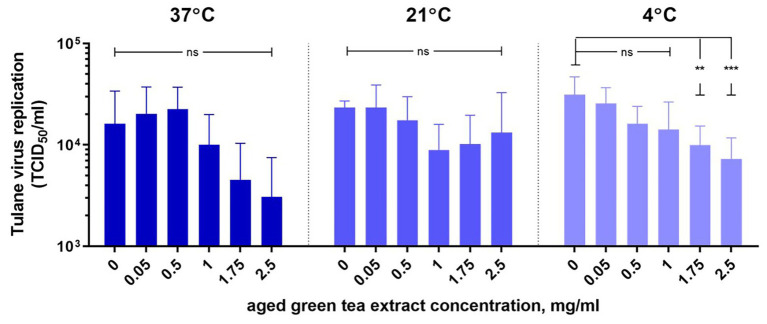
Inactivation of Tulane virus with aged-green tea extract in suspension. Tulane virus (4.02 ± 0.2 log10 TCID_50_/ml) was either treated or not treated with aged-green tea extract at concentrations up to 2.5 mg/ml for 1 h at 37, 21, and 7°C. Samples were diluted 1:1 v/v with Opti-MEM to neutralize aged-green tea extract. The mean of each data point is shown and error bars indicate SD of three experiments with three technical replicates for each treatment and time point. For each sample, we performed one-way ANOVA followed by Dunnett’s multiple comparisons test. *p* are compared with the nontreated sample: ^**^p < 0.01; ^***^p < 0.001; ns, not significant.

**Figure 4:**
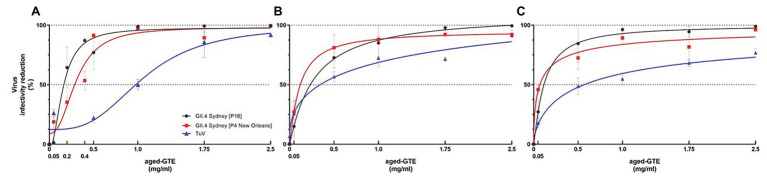
Effective concentration (EC_50_) of aged-GTE that inhibits human norovirus and Tulane virus replication. Viruses were exposed to aged-GTE for 1 h at 37°C **(A)**, 21°C **(B)**, and 7°C **(C)**. A nonlinear regression (curve fit) function on data sets of three experiments with three technical replicates for each treatment, time point, and virus were used to obtain EC_50_ values. The data is based on genomic copies for human norovirus and on TCID_50_ for Tulane virus.

HIE monolayers pretreated with aged-GTE at different temperatures showed complete inhibition of human norovirus replication at concentrations as low as 1.0 mg/ml for 37°C, 1.75 mg/ml for 21°C, and 2.5 mg/ml for 7°C (Figure 2). Tulane virus showed 0.85, 0.75, and 0.65 log TCID_50_/ml reduction when exposed to GTE at 2.5 mg/ml at 37, 21, and 7°C, respectively (Figure 3). The effective concentration (EC_50_) resulted as 0.16 and 0.31 mg/ml aged-GTE tested at 37°C, 0.26 and 0.16 mg/ml at 21°C, and 0.12 and 0.21 at 7°C for human norovirus GII.4 Sydney[P16] and GII.4 Sydney[P4 New Orleans], respectively (Figure 4).

Based on cell infectivity, Tulane virus EC_50_ was 1.0 mg/ml at 37°C, 0.34 mg/ml at 21°C, and 0.45 mg/ml at 7°C (Figure 4). By RT-qPCR, titers of Tulane virus exposed to GTE at 2.5 mg/ml at 37, 21, and 7°C dropped 1.37, 0.97, and 0.65 log genomic copies/ml (Figure 3). Of note, Tulane virus was not completely inactivated by GTE even at 2.5 mg/ml, regardless of temperature. Given the cytotoxicity on LLC-MK2 cells by aged-GTE at concentration higher than 2.5 mg/ml, we were unable to define at what GTE concentration Tulane virus would be completely inactivated.

## Discussion

Using the recently reported HIE cell culture system for human norovirus, we demonstrated *in vitro* that aged-GTE completely inhibited human norovirus GII.4 infectivity. Interestingly, infectious Tulane virus, which has been described as one of the most promising surrogate viruses for human norovirus (Cromeans et al., 2014), was only moderately reduced by aged-GTE indicating that this virus is more resistant than human norovirus to inactivation by aged-GTE.

We observed minor differences between the inactivation patterns of GII.4 Sydney[P16] compared to GII.4 Sydney[P4 New Orleans], which are likely caused by unknown factors in norovirus stool filtrates (Costantini et al., 2018; Estes et al., 2019).

Previous studies have suggested that GTE and catechins most likely inactivate viruses by nonspecific binding with the virus receptors thereby preventing the virus to bind to the host cells (Friedman, 2007; Oh et al., 2013; Steinmann et al., 2013; Calland et al., 2015; Gómez-Mascaraque et al., 2016; Falcó et al., 2019). Inactivation data for other viruses show EC_50_ values for adenovirus of 0.1–1.4 mg/ml (Weber et al., 2003), hepatitis C virus of 4 μg/ml (Calland et al., 2015), and FCV of 12 mg/ml (Oh et al., 2013).

Most studies have assessed the antiviral activity of GTE and aged-GTE using cultivatable surrogate viruses (Oh et al., 2013; Steinmann et al., 2013; Randazzo et al., 2017; Falcó et al., 2019). In general, our HIE data are in agreement with studies that used FCV, MNV, or HAV. For example, both HAV and MNV were completely inactivated by GTE at 37°C (Randazzo et al., 2017), while others reported reduced infectivity of FCV after exposure to catechins at 25°C (Oh et al., 2013; Su and D’Souza, 2013; Seo et al., 2016).

We included Tulane virus for comparative purposes as this virus has been shown as one of the most promising surrogate viruses to assess inactivation and disinfection treatments against human norovirus (Cromeans et al., 2014; Drouaz et al., 2015), and recent HIE data showing the lack of alcohols to inactivate human norovirus resembled the inactivation patterns of Tulane virus reported previously (Cromeans et al., 2014; Costantini et al., 2018). However, Tulane virus infectivity was reduced less than 1 log_10_ by aged-GTE compared with complete inactivation of human norovirus in the HIE system. Generally, similar inactivation levels have been recorded for Tulane virus by RT-qPCR compared to infectivity in cell culture, unlike previous findings based on MNV and FCV exposed to thermal and chlorine treatments (Knight et al., 2016).

Our study targets the debate on the suitability of surrogates to infer human norovirus behavior in inactivation experiments of interest for antiviral development, food safety protocols, and environmental decontamination (Richards, 2012). Our data showed that Tulane virus failed to closely mimic the pathogen it represents, being more resilient than human norovirus GII.4 when exposed to aged-GTE. On a practical perspective and given the low infectious dose of the human pathogen, the use of the most resistant surrogate could represent a benchmark for tuning the operational practices to prevent and control human norovirus in food and environmental settings, especially when the adoption of a presence/absence criterion has been proposed as the most appropriated (Koopmans and Duizer, 2004; Lowther et al., 2012; Suffredini et al., 2014; Bosch et al., 2018). Beside the potential over regulation and increased production cost, the approach of using conservative surrogates as proof-of-principle testing coupled to validation on HIEs spares the need of conducting human challenge studies as earlier proposed (Richards, 2012).

Our study has several limitations. Unlike surrogate viruses which can be grown to high viral titers, the maximum increase in replication of human norovirus is between 2 and 3 log_10_, while typical inactivation patterns of at least 3–5 log_10_ are used for other pathogens (US EPA, 2018). Moreover, relative high amounts of virus (10^5^ genome equivalents) are used for the HIE system, which is significantly higher than the amount needed to cause human infection and the amount of virus that is typically detected in environmental samples. Therefore, increasing the sensitivity of the HIE system should be explored. In addition, strain-specific replication requirements such as bile acids have been identified for several non-GII.4 genotypes (Murakami et al., 2020). This is an important area of future research as different inactivation patterns against different human norovirus strains have been reported (Park et al., 2010). Finally, the HIE model is costly and labor intensive and additional improvements are ultimately required to use this system more widely including for applied virology (Costantini et al., 2018; Manuel et al., 2018; Chan et al., 2019; Estes et al., 2019).

These limitations hampered further evaluation of presumed active ingredients and their potential synergistic effects since more than nine catechins and derivatives have been reported as responsible of the antiviral activity (Falcó et al., 2017).

In future studies, the HIE model could be used to validate the performance of alternative and more affordable methods such as *in situ* capture RT-qPCR (Tian et al., 2018; Falcó et al., 2019) and viability RT-qPCRs (Randazzo et al., 2016; Chen et al., 2020) to assess the infectivity of a given sample exposed to inactivation treatments.

In conclusion, we confirmed the antiviral activity of GTE at different temperatures and expanded this observation by showing not only reduction on viral genome copies, but also demonstrate that aged-GTE completely inactivates human norovirus GII.4 replication in a laboratory setting. In addition, we have showed that Tulane virus could likely be used as a conservative surrogate for measuring inactivation of norovirus with natural compounds.

## Data Availability Statement

All datasets presented in this study are included in the article/supplementary material.

## Author Contributions

All authors listed have made a substantial, direct and intellectual contribution to the work, and approved it for publication.

### Disclaimer

The findings and conclusions in this article are those of the authors and do not necessarily represent the official position of the Centers for Disease Control and Prevention.

### Conflict of Interest

EM was employed by Cherokee Nation Assurance.

The remaining authors declare that the research was conducted in the absence of any commercial or financial relationships that could be construed as a potential conflict of interest.
